# Regeneration of Mature Norway Spruce Stands: Early Effects of Selective Cutting and Clear Cutting on Seepage Water Quality and Soil Fertility

**DOI:** 10.1100/tsw.2001.327

**Published:** 2001-11-10

**Authors:** Wendelin Weis, Christian Huber, Axel Gattlein

**Affiliations:** Forest Nutrition and Water Resources, Technische Universität München, Freising, Germany

**Keywords:** forest management, regeneration, Norway spruce, nitrate concentration, water quality, soil fertility

## Abstract

The cutting of trees influences element turnover in the forest ecosystem. The reduction of plant uptake, as well as an increased mineralization and nitrification due to higher soil temperature and soil moisture, can lead to considerable losses of nutrients from the main rooting zone. This may result in a reduced soil fertility and a decrease in drinking water quality due to high nitrate concentrations in the seepage water. In Bavaria (Germany) selective cutting is preferred to clear cutting when initiating the regeneration of Norway spruce stands with European beech. This paper summarizes the early effects of both forest management practices on soil fertility and seepage water quality for three different sites. Shown are the concentrations of nitrogen and base cations in the seepage water as well as the water and ion fluxes during the first year after tree cut. Nutrient inputs decreased on thinned plots and even more at clear-cuts. Nitrate concentrations in the seepage water are hardly affected by moderate thinning; however, on clear-cuts, the nitrate concentration increases significantly, and base cations are lost from the upper mineral soil. This effect is less obvious at sites where a dense ground vegetation, which is able to take up excess nitrogen, exists.

## INTRODUCTION

In Germany, and especially in Bavaria, official forest management on public forest land aims at the preservation and re-establishment of stable and productive mixed forests in accordance with the specific site conditions. As a consequence, wherever possible, mature Norway spruce stands (*Picea abies* (L.) Karst) are regenerated with European beech (*Fagus sylvatica* L.) and other broadleaf species[1].

In practice young beech plants (ca. 5 years old) are planted after a more-or-less intense thinning of the mature spruce stand if no natural regeneration with beech occurs. Thinning influences the input of water and elements as well as microclimatic conditions, the uptake of nutrients, and the development of the ground vegetation. As a consequence, the element cycling in the soil is altered. In nitrogen-saturated systems there may be high losses of nitrogen from the ecosystem during regeneration; which affect the seepage water and soil quality due to nitrate leaching and, thus, induced output of nutrient cations. In the long run this may lead to less fertile soils and nutritional deficiencies in the young trees.

In this paper we compare nutrient cycling and element losses from stands of Norway spruce regenerated with European beech during the first year after selective cutting and clear cutting. For information about the impact of site characteristics, the experimental plots were established at three different sites, all of them representative for a substantial part of Bavarian forests. At all sites, long-term monitoring plots in untreated, mature Norway spruce stands serve as references.

## EXPERIMENTAL METHODS

In 1999 the experimental plots were installed at the three sites: Ebersberg, Flossenbürg, and Höglwald. All sites are characterized by a strongly acidified upper mineral soil but differ in stand density, climate, element input, parent material, and soil (Tables 1 and 2).

- At Ebersberg, some 30 km east of Munich, the soil is rich in base cations in the lower mineral soil due to calcareous and dolomitic gravel deposits from the last glacial period. The moderate nitrogen and sulfur input is typical for most of the forests in southern Bavaria.
- The site near Flossenbürg is situated in the inner Oberpfälzer Wald at the Czech border. The soil is rather poor in base cations. The site suffers high inputs of nitrogen and sulfur. Because of the elevation, the mean annual temperature is rather low as compared to the other sites.
- Höglwald, located between Munich and Augsburg, is a long-term ecological monitoring and experimentation site where element cycling in spruce and beech stands has been studied for nearly 2 decades. The site is excellently supplied with nutrients; however, high nitrogen input, especially from agricultural sources, and a high potential for nitrification in the soil cause high nitrate concentrations in seepage water under mature Norway spruce. Here, regeneration may lead to even higher nitrate losses from the soil due to increasing mineralization; thus, regeneration represents a potential ecological risk. For details about the stand and site characteristics and some of the experiments carried out at the Hoeglwald see Kreutzer[2], Kreutzer et al.[3], and Kreutzer and Weiss[4].

At each of the three sites a small clear-cut (about 0.5 ha) and a selective cutting (30 × 30 m), removing 20 to 30% of basal area, were carried out during winter 1999–2000. Stems including bark were removed from the forest, and slash remained at the site, although not at the investigation plots. At the clear-cut and in the thinned spruce stand, 5-year-old trees of European beech were planted early in spring 2000. On all plots, infiltration and seepage water under the organic layer (gravitation lysimeters) and in 40-cm soil depth (suction cup lysimeters) were sampled in intervals ranging from 10 to 30 days with ten replicates each. At each site, a nearby long-term monitoring plot in a mature spruce stand was taken as a control plot. Because the sampling started in July 1999, well before tree cut, induced changes can be well documented.

The water samples were analyzed for pH, the concentrations of $Cl^-$, $NO_3^-$, $SO_4^{2-}$, and $H_2PO_4^-$ (IC2010i, Dionex); and the cations $NH_4^+$ (SKALAR SUN plus system, SKALAR analytic GmbH), $Al^{3+}$, $Fe^{3+}$, $Mn^{2+}$, $Ca^{2+}$, $Mg^{2+}$, $K^+$, and $Na^+$ (ICO-OES, Perkin Elmer). The water fluxes were measured either directly (throughfall) or using the water budget model "Höglwald"[5]. The element fluxes were calculated by multiplying the element concentrations with daily water fluxes. Additionally, the development and the nutritional status of the ground vegetation and the young beech trees were monitored in order to estimate the element fluxes into the vegetation.

**TABLE 1**
**Characteristics of the Three Experimentation Sites: Ebersberg, Flossenbürg, and Höglwald**

| Climate | altitude | mean temperature | precipitation | N-input bulk |
|---|---|---|---|---|
| | [m] | [°C] | [mm a$^{-1}$] | [kg ha$^{-1}$] |
| Ebersberg | 540 | 7.5 | 1000 | 13 |
| Flossenbürg | 840 | 5.5 | 900 | 19 |
| Höglwald | 540 | 7.8 | 870 | 31 |

| Soil | humus type | soil type | soil texture |
|---|---|---|---|
| Ebersberg | raw humus - moder | parabrown earth | loamy silt - sandy loam |
| Flossenbürg | raw humus - grass-root- moder | podsolic brownearth | sandy loam - loamy sand |
| Höglwald | typical moder | parabrown earth | sandy loam - clay loam |

| Stand | age | number of trees | mean DBH | basal area | mean heigt | growing stock |
|---|---|---|---|---|---|---|
| | [a] | [ha$^{-1}$] | [cm] | [m$^2$ha$^{-1}$] | [m] | [m$^3$] |
| Ebersberg | 85 | 520 | 40 | 64 | 33 | 966 |
| Flossenbürg | 85-100 | 544 | 31 | 41 | 25 | 560 |
| Höglwald | 95 | 620 | 39 | 79 | 35 | 1309 |

**TABLE 2**
**Soil Characteristics of the Three Experimentation Sites: Ebersberg, Flossenbürg, and Höglwald**

| | pH ($H_2O$) | C [%] | N | C/N | CEC [µmol$_c$ g$^{-1}$] | BS % |
|---|---|---|---|---|---|---|
| **Ebersberg** | | | | | | |
| LOF | 4.3 | 46.0 | 1.8 | 25.5 | 221.8 | 77.3 |
| OH | 3.9 | 37.8 | 1.9 | 20.2 | 200.0 | 59.8 |
| 0-5 cm | 3.6 | 11.3 | 0.6 | 19.5 | 153.4 | 8.8 |
| 5-20 cm | 4.1 | 3.4 | 0.2 | 17.7 | 92.5 | 4.9 |
| 20-40 cm | 4.3 | 1.3 | 0.1 | 13.5 | 70.4 | 5.1 |
| **Flossenbürg** | | | | | | |
| LOF | 3.9 | 42.0 | 1.9 | 22.1 | 183.6 | 52.2 |
| OH | 3.4 | 36.4 | 1.7 | 21.8 | 208.9 | 17.9 |
| 0-5 cm | 3.2 | 20.5 | 0.9 | 22.1 | 158.2 | 11.7 |
| 5-20 cm | 3.5 | 5.3 | 0.2 | 22.4 | 129.9 | 4.1 |
| 20-40 cm | 4.2 | 2.3 | 0.1 | 19.3 | 64.1 | 3.4 |
| **Höglwald** | | | | | | |
| LOf | 4.0 | 46.1 | 1.6 | 28.2 | 227.0 | 70.4 |
| Oh | 3.5 | 39.3 | 1.5 | 25.5 | 201.9 | 48.3 |
| 0-3 cm | 3.6 | 9.1 | 0.4 | 20.6 | 164.8 | 20.2 |
| 3-10 cm | 3.7 | 3.3 | 0.2 | 21.5 | 56.4 | 4.2 |
| 10-20 cm | 4.1 | 0.7 | 0.0 | 22.3 | 57.3 | 4.8 |
| 20-40 cm | 4.1 | 0.5 | 0.0 | 14.4 | 111.9 | 16.2 |

*Note*: CEC was determined after extraction with 1 *M* $NH_4Cl$.

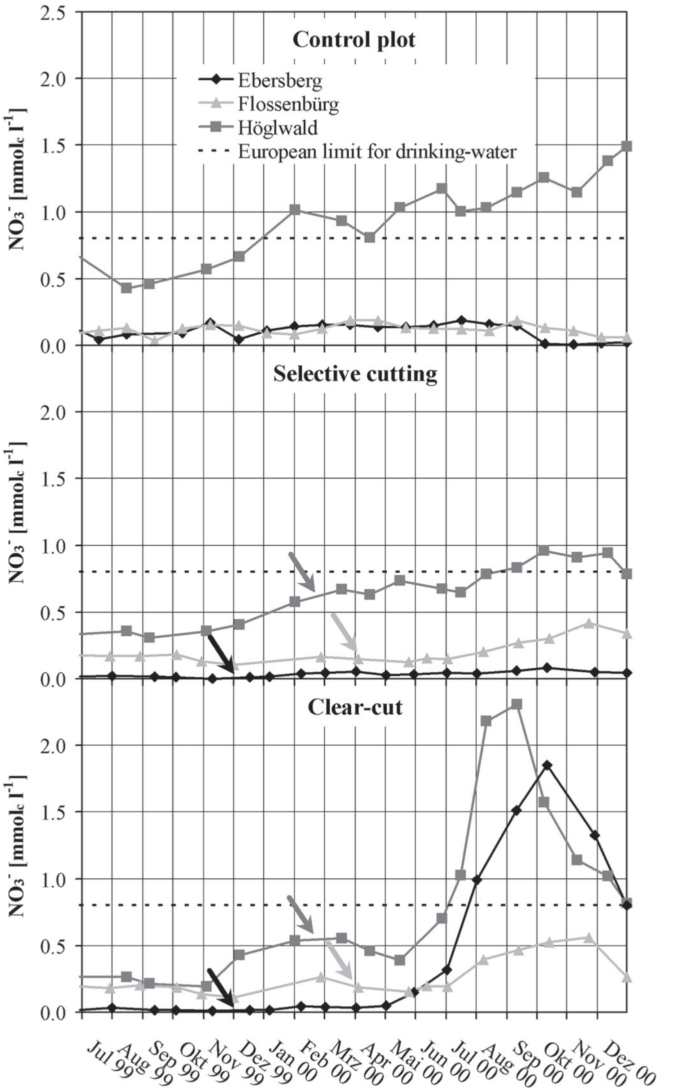

**FIGURE 1.** Mean nitrate concentration of control, selective-cutting, and clear-cut plots in Ebersberg, Flossenbürg, and Höglwald (40-cm soil depth). Cutting was done between December 1999 and April 2000 (see arrows).

## RESULTS AND DISCUSSION

The time courses in Fig. 1, beginning some 6 months before and ending about 1 year after the felling of the trees, show only a slight increase of nitrate concentrations in the seepage water after *selective cutting* (Fig. 1). At Ebersberg and Höglwald, where from the beginning of our study, the control plots showed higher nitrate levels compared to the manipulated plots, the nitrate concentrations after selective cutting are comparable to or even lower than the corresponding concentration from the control plots; however, the impact of selective cutting on nitrate concentration is clearly visible at Flossenbürg, where the thinning was more concentrated to the center of the plot. At Ebersberg, too, some of the suction cups, situated mostly but not always near a cut tree, show higher nitrate concentrations; however, these concentrations were never as high as on the clear-cut plot (Fig. 2). An increase in nitrate output after selective cutting has already been observed in beech forests in Germany[6,7].

On the plots where *clear-cuts* were carried out, a clear effect on the mean nitrate concentration in 40-cm soil depth is visible only at Höglwald and Ebersberg (Fig. 1). Concentrations well above the European limit for drinking water (50 mg $l^{-1}$) occur at the end of the first vegetation period after the clear-cut. The nitrate in 40-cm depth is accompanied mostly by aluminum because the base saturation in the upper mineral soil is low; however, calcium and especially magnesium are removed from the upper soil as well (Fig. 3). Similar results were reported from a number of forest ecosystem studies on scales ranging from single plots to whole catchments[8,9,10,11]. At Flossenbürg, an increase in nitrate concentration is also visible, but on a much lower level. Here the existing ground vegetation, mostly *Vaccinium myrtillus* and *Deschampsia flexuosa*, is able to partly take up excess nitrogen. At spots with a denser cover of vegetation, the nitrate concentrations in seepage water seem to be unaffected by the removal of the trees, whereas at spots where no or sparse ground vegetation is present, nitrate concentrations show the expected rise after the removal of the mature trees (Fig. 4).

In order to estimate whether the cutting of trees during the regeneration of spruce forests affects the availability of nutrients, we calculated *element fluxes* from the measured concentrations and modeled water fluxes (Table 3). After a partial or complete removal of the canopy due to tree felling, interception loss and stand transpiration decrease accordingly. As a consequence, compared to the control plots, the plots with selective cutting—especially the clear-cut plots—show higher water fluxes into the soil and in 40-cm soil depth; however, dry deposition and leaching from the canopy are reduced, resulting in lower inputs of nitrogen, sulfur, potassium, calcium, and magnesium.

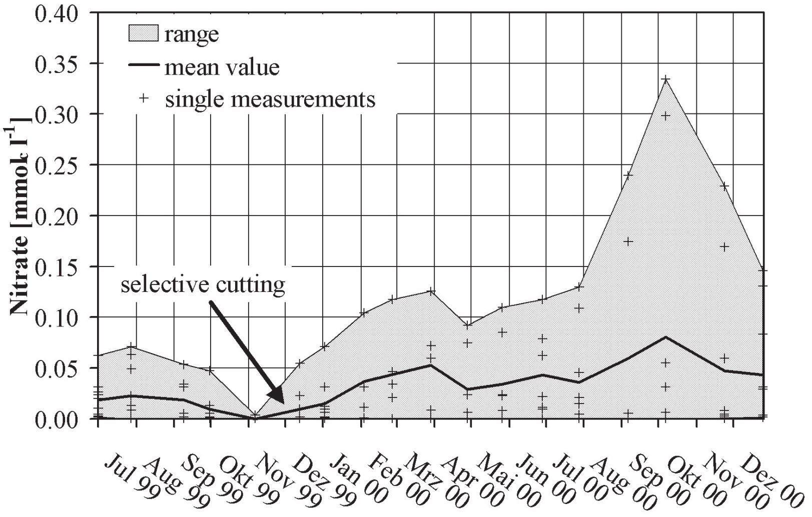

**FIGURE 2.** The range of nitrate concentrations in 40-cm soil depth after selective cutting in Ebersberg.

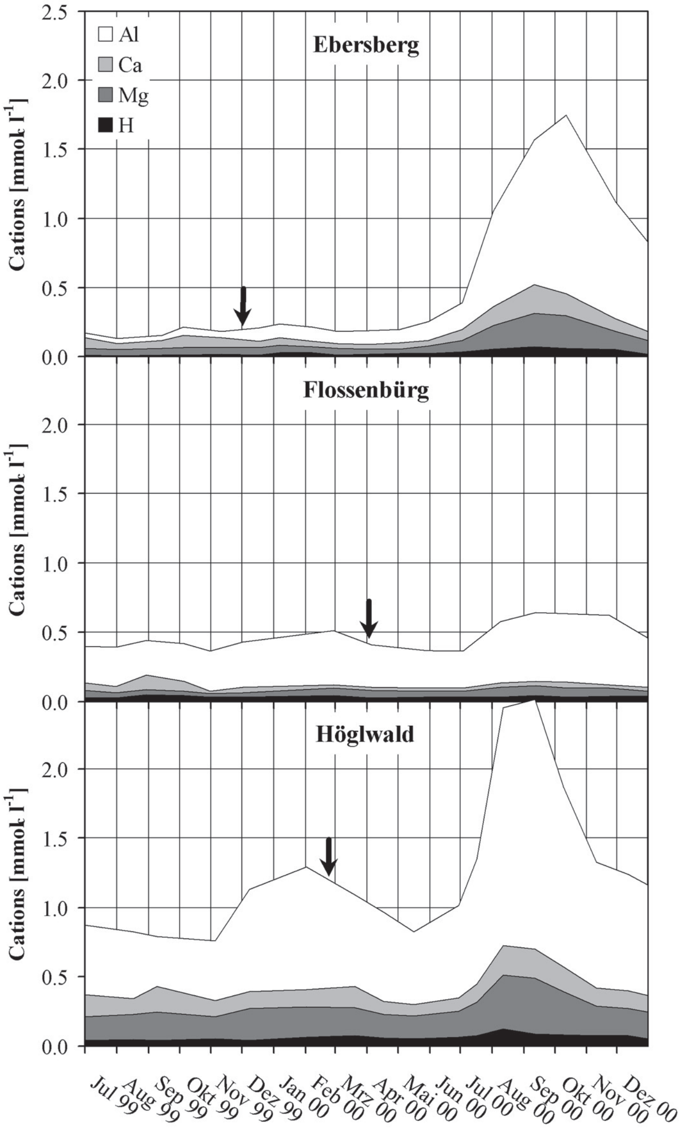

**FIGURE 3.** Cation concentrations of the clear-cut plots in Ebersberg, Flossenbürg, and Höglwald (40-cm soil depth). Cutting was done between December 1999 and April 2000 (see arrows).

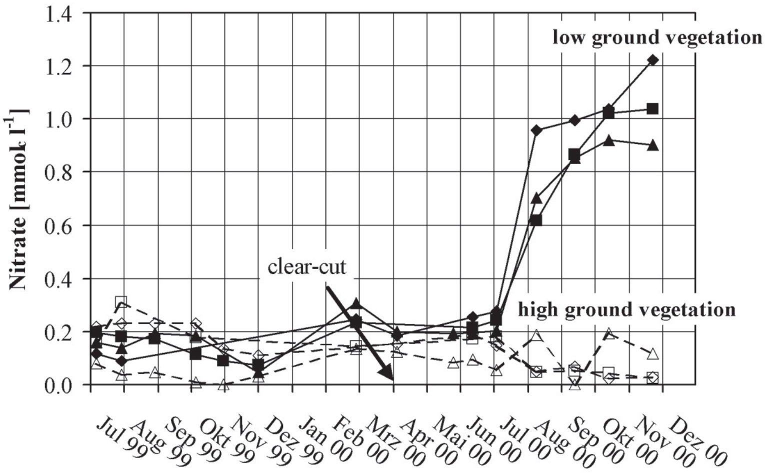

**FIGURE 4.** The influence of the ground vegetation on nitrate concentration in 40-cm soil depth at the clear-cut plot in Flossenbürg.

## TABLE 3
## Annual Water and Ion Fluxes for Control, Selective-Cutting, and Clear-cut Plots in Ebersberg, Flossenbürg, and Höglwald

| Site/Plot | Sample | Flux | $Cl^-$ | $NO_3^-$ | $SO_4^{2-}$ | $NH_4^+$ | $H^+$ | $Al^{3+}$ | $Fe^{3+}$ | $Mn^{2+}$ | $Ca^{2+}$ | $Mg^{2+}$ | $K^+$ | $Na^+$ |
|---|---|---|---|---|---|---|---|---|---|---|---|---|---|---|
| | | [mm] | [mmol$_c$ m$^{-2}$] | | | | | | | | | | | |
| **Ebersberg** | | | | | | | | | | | | | | |
| Control plot | Throughfall | 922 | 32 | 83 | 35 | 76 | 7 | 0 | 3 | 4 | 51 | 17 | 48 | 14 |
| | Flux 40cm | 586 | 36 | 58 | 209 | 0 | 2 | 102 | 2 | 32 | 73 | 48 | 27 | 30 |
| Selective cutting | Throughfall | 975 | 18 | 55 | 30 | 54 | 11 | 0 | 3 | 2 | 32 | 10 | 26 | 9 |
| | Flux 40cm | 750 | 20 | 38 | 134 | 0 | 11 | 55 | 2 | 9 | 46 | 36 | 12 | 30 |
| Clear-cut | Throughfall | 1118 | 9 | 37 | 27 | 42 | 35 | 0 | 3 | 0 | 18 | 3 | 3 | 11 |
| | Flux 40cm | 1102 | 41 | 815 | 130 | 0 | 42 | 593 | 3 | 58 | 103 | 132 | 16 | 58 |
| **Flossenbürg** | | | | | | | | | | | | | | |
| Control plot | Throughfall | 688 | 27 | 62 | 60 | 55 | 15 | 0 | 2 | 2 | 38 | 12 | 43 | 15 |
| | Flux 40cm | 424 | 18 | 56 | 150 | 0 | 21 | 176 | 1 | 2 | 14 | 13 | 6 | 13 |
| Selective cutting | Throughfall | 739 | 14 | 44 | 40 | 52 | 19 | 0 | 2 | 0 | 17 | 5 | 21 | 12 |
| | Flux 40cm | 587 | 18 | 126 | 149 | 0 | 21 | 235 | 2 | 1 | 10 | 21 | 3 | 20 |
| Clear-cut | Throughfall | 805 | 14 | 52 | 37 | 46 | 20 | 0 | 2 | 0 | 13 | 4 | 9 | 14 |
| | Flux 40cm | 775 | 28 | 245 | 128 | 0 | 28 | 299 | 3 | 3 | 21 | 43 | 6 | 34 |
| **Höglwald** | | | | | | | | | | | | | | |
| Control plot | Throughfall | 613 | 20 | 77 | 47 | 133 | 3 | 0 | 2 | 4 | 31 | 14 | 43 | 10 |
| | Flux 40cm | 298 | 19 | 333 | 182 | 0 | 24 | 274 | 1 | 39 | 84 | 100 | 9 | 39 |
| Selective cutting | Throughfall | 647 | 18 | 66 | 39 | 112 | 2 | 0 | 2 | 3 | 31 | 12 | 40 | 8 |
| | Flux 40cm | 381 | 19 | 307 | 171 | 0 | 24 | 254 | 1 | 25 | 79 | 87 | 13 | 37 |
| Clear-cut | Throughfall | 919 | 11 | 37 | 34 | 58 | 5 | 0 | 3 | 1 | 18 | 5 | 9 | 7 |
| | Flux 40cm | 901 | 46 | 999 | 366 | 0 | 69 | 911 | 2 | 37 | 129 | 228 | 30 | 70 |

As the buffering capacity of the canopy surface decreases, the direct proton input slightly increases.

The element fluxes in 40-cm soil depth show no increase for nitrate at the selective cut plots at Ebersberg and Höglwald, whereas they double at Flossenbürg. A possible explanation is the higher level of nitrate output from the control plots at Ebersberg and Höglwald and the comparatively more centered thinning at Flossenbürg. Clear-cuts at all sites resulted in a high nitrate output. Compared to the control plots, we observe a more than tenfold increase at Ebersberg, a fivefold increase at

Flossenbürg, and a threefold increase at Höglwald; however, the latter site shows high nitrate losses from the control plot as well; consequently, at Höglwald the nitrate output from the clear-cut plot is the highest of all three sites.

The high nitrate losses from the upper mineral soil under the clear-cut plots lead to high outputs of aluminum and significant losses of calcium, magnesium, and potassium at the sites rich in base cations. For the Höglwald, a *nutrient balance* was calculated for the first year after clear-cut and compared to the control plot (Table 4). Listed in Table 4 are the differences between clear-cut plot and control plot (clear-cut *minus* control) for the fluxes of nitrogen, potassium, magnesium, and calcium. "Infiltration" signifies the element input into the soil including the amount of nutrients leached from the canopy. "Canopy litter" is the annual net input of nutrients with canopy litterfall into the soil. Assuming that the yearly nutrient uptake of the trees for canopy growth equals litterfall (steady-state needle mass), this input is zero for the control plot. The same holds true for "fine root litter," where the nutrient input into the soil of the clear-cut plot due to root mineralization is set equal to the entire amount of nutrients in the roots with a diameter less than 2 mm. "Increment" is the nutrient uptake for stem growth, which is zero on the clear-cut plot. "Flux 40 cm" is derived from the element fluxes listed in Table 3. A negative nutrient balance indicates that the loss of a respective nutrient from the soil is higher at the clear-cut plot than at the control plot. In turn, the nutrient status of the system decreases. This is the case for nitrogen, potassium, and magnesium, but not for calcium; however, these nutrient losses do not seem to be essential when compared to the nutrient supply in the upper 40 cm of the soil (Table 5). Even for magnesium, which seems to be the most critical element, the surplus loss from the clear-cut plot amounts to less than 15% of the available magnesium supply.

So far, the results from our studies cover only the first year after tree cut; however, we have to consider that, when using selective cutting, the regeneration of mature spruce forests with beech needs further thinning during the next 2 decades. Each thinning may possibly lead to increased nutrient outputs with the seepage water; thus, a final comparison of the nutrient losses from spruce forests regenerated with beech via selective cutting and clear-cut is not yet possible.

**TABLE 4**
**Differences in Nutrient Fluxes and Nutrient Losses from the Top 40 cm of Soil for the Clear-Cut Plot at Höglwald Compared to the Control Plot**

| | N | K | Mg | Ca |
|---|---|---|---|---|
| | [mmol m$^{-2}$] | [mmol$_c$ m$^{-2}$] | | |
| throughfall | -115 | -33 | -9 | -13 |
| canopy litter | 489 | 27 | 32 | 171 |
| fine root litter | 118 | 9 | 14 | 29 |
| **sum input** | **492** | **3** | **38** | **187** |
| increment | -53 | -5 | -7 | -25 |
| flux 40cm | 666 | 21 | 128 | 45 |
| **sum output** | **614** | **16** | **121** | **20** |
| ***balance*** | ***-122*** | ***-13*** | ***-83*** | ***167*** |

*Note*: Clear-cut *minus* control.

**TABLE 5**
**Element Storage in the Upper 40 cm of the Soil at Höglwald**

| Site | C | N | H | Al | Ca | Fe | K | Mg | Mn | Na |
|---|---|---|---|---|---|---|---|---|---|---|
| | [g m$^{-2}$] | | [mmol$_c$ m$^{-2}$] | | | | | | | |
| Ebersberg | 8555 | 515 | 439 | 22381 | 617 | 425 | 414 | 336 | 529 | 367 |
| Flossenbürg | 21885 | 959 | 1729 | 33574 | 978 | 5066 | 431 | 552 | 239 | 254 |
| Höglwald | 8780 | 521 | 1086 | 27014 | 923 | 816 | 529 | 648 | 656 | 291 |

*Note*: C and N are total supply; cation supply was calculated from exchangeable cations.

## CONCLUSIONS

Selective thinning as an initializing procedure for the regeneration of spruce forests has little effect on the water and element balance of the ecosystem during the first year after the thinning. Clear-cut, however, clearly affects the nitrate concentration in the seepage water, which reaches values well above the European limit for drinking water (50 mg $l^{-1}$) when no significant ground vegetation is present. Together with nitrate, primarily aluminum, but also potassium, magnesium, and calcium are leached from the upper mineral soil where the base saturation is low; however, during the first year after clear-cut the losses of base cation nutrients are not fatal for the ecosystem when compared to the supply of available cations.

## ACKNOWLEDGMENTS

We would like to thank the Bavarian State Ministry for Agriculture and Forests and the Federal Ministry of Education and Research for their financial support. Our thanks belong also to the Bavarian State Institute of Forestry for their ready supply of data from the forest monitoring plots in Ebersberg and Flossenbürg.

| | | |
|---|---|---|
| Received: | July | 25, 2001 |
| Revised: | October | 18, 2001 |
| Accepted: | October | 25, 2001 |
| Published: | November | 10, 2001 |
